# Non-channeled Video Laryngoscopy as an Alternative to Conventional Laryngoscopy for Intubating Adult Patients in the Intensive Care Unit

**DOI:** 10.7759/cureus.40716

**Published:** 2023-06-21

**Authors:** Aparna Shukla, Ravi Shanker, Vipin K Singh, Gyan Prakash Singh, Tanushree Srivastava

**Affiliations:** 1 Anaesthesiology, King George’s Medical University, Lucknow, IND; 2 Anaesthesiology, Integral Institute of Medical Sciences and Research, Lucknow, IND

**Keywords:** video laryngoscope, intensive care unit (icu), video laryngoscopy (vl), macintosh laryngoscope, conventional laryngoscopy, intubation

## Abstract

Background

Endotracheal intubation in the intensive care unit (ICU) is often a risky procedure due to the emergency situation, unstable condition of the patient, and technical problems such as inadequate positioning. Several new techniques, such as video laryngoscopy, have been developed recently to improve the success rate of first-pass intubations and reduce complications. We conducted this study to compare a non-channeled reusable video laryngoscope BPL VL-02 (manufactured by BPL Medical Technologies, Bangalore, India) with a conventional laryngoscope for intubation of adult patients in the ICU.

Methodology

A total of 72 ICU patients were randomly allocated to be intubated with either conventional direct laryngoscopy via Macintosh blade (group A) or video laryngoscopy with BPL VL-02 (group B). All patients were intubated by the primary investigator and the assistant noted the following parameters: the total number of intubation attempts, total duration of intubation, assistance or alternative technique required, Cormack Lehane grading, and any complications.

Results

There was no significant difference in the Cormack Lehane grading, number of attempts, or complications between the two groups. On comparing the assistance required during intubation in patients, it was observed that four (11.11%) patients in group A and seven (19.44%) patients in group B needed backward, upward, and rightward pressure on the larynx assistance during intubation. In five (13.89%) patients in group B, Stylet was required during intubation. The difference was statistically significant (p = 0.0308). The video laryngoscopy group (group B) had a longer mean duration of intubation (64.36 ± 6.28 seconds) compared to group A (45.72 ± 11.45 seconds), and the difference was statistically significant (p < 0.0001).

Conclusions

Non-channeled video laryngoscope (BPL VL-02) is not a suitable alternative to conventional direct laryngoscopy with a Macintosh blade in terms of successful first-pass intubation, total duration of intubation, and assistance required.

## Introduction

Endotracheal intubation with conventional laryngoscopy (CL) is the standard technique for establishing the airway. Despite the unpredictability and urgency of emergency intubation, the success rate of CL is above 85% [1]. Emergency intubation carries a greater risk of procedure-related complications and unfavorable outcomes, such as aspiration, hypoxia, and mortality, compared to elective intubation [2]. In recent years, video laryngoscopes (VLs) have risen in popularity as they may improve the success rate of first-pass intubation and reduce complications [3,4]. VLs are relatively new intubation devices that offer an image of the glottis via a video camera or video chip mounted on the laryngoscope blade tip. There are three fundamental types, namely, those with a standard Macintosh-shaped blade, those with an angled blade, and those with a tube passage channel. Each design has its own advantages and disadvantages [5,6].

The VL needs less force and less manipulation of the cervical spine. The video camera of the VL eliminates the necessity for alignment of the three airway axes, resulting in improved glottic vision. VL use has been proven to enhance first-pass intubation success rates in patients with difficult airways, in addition to obstetric, pediatric, and trauma patients, especially with an unstable cervical spine [7-11].

The shared airway view provided by a VL enables multiple people to view the airway simultaneously, which is invaluable in a teaching environment, and it also improves team coordination during difficult airway management. The visual confirmation of tracheal tube placement greatly increases the safety margin of airway management. By preventing close contact with the airway, as was the case with patients infected with coronavirus disease 2019 (COVID-19), the use of a VL reduces exposure risk from infected patients [12,13]. But do these advantages translate to better results when attempting intubations? We tried to assess that in our study for one particular type of VL (BPL VL-02; manufactured by BPL Medical Technologies, Bangalore, India).

We hypothesized that VL has no advantage compared to CL in terms of success and time taken for intubation. The primary objective was to compare the percentage of successful first-pass endotracheal intubation between both groups. The secondary objectives were to compare the time taken for successful intubations, the percentage of successful orotracheal intubation at the second attempt, compare the Cormack-Lehane grading of glottic visibility [14], the percentage of patients requiring alternative techniques or assistance for intubation, and to assess complications, such as trauma, hypoxemia, and esophageal intubation.

## Materials and methods

Study design

This was a prospective randomized controlled trial conducted on 72 adult patients in an intensive care unit (ICU) over a period of one year. Approval of the Institutional Ethics Committee (IV-PGTSC-IIA/P34) and CTRI approval (CTRI/2022/06/043436) were obtained before commencing the study.

Sample size calculation

The sample size was calculated based on a previous study conducted by Yi et al. [15] using the Fleiss formula [16] with a power of 90% and a 95% confidence interval. The odds ratio was 11.92 (odds ratio of predicted difficult video laryngoscopy versus direct laryngoscopy for adult patients). The risk/prevalence ratio was 37.7, and the risk/prevalence difference was 34 (Table 1).

**Table 1:** Sample size calculation.

Two-sided significance level (1-alpha)	95
Power (1-beta, % chance of detecting)	90
Ratio of sample size, unexposed/exposed	1
Percent of unexposed with the outcome	5
Percent of exposed with the outcome	39
Odds ratio	11.92
Risk/Prevalence ratio	37.7
Risk/Prevalence difference	34
Total sample size	72

Randomization

A statistician who had no role in patient recruitment generated a randomization sequence for study participants on the computer in blocks of four. Patients were allotted to either of the two following groups: group A (intubated with CL using a Macintosh blade) and group B (intubated with VL using BPL VL-02). Implementation of the random allocation was done by an assistant who gave the laryngoscope to the investigator. Parameters were also recorded by the assistant as an electronic report. Software used to collect this data automatically allocated the patients, thereby ensuring concealment. Blinding was only done at the analysis level where the statistician analyzing the results was unaware of which group belonged to CL or VL.

Patient selection criteria

Adult males and females over 18 years of age with Mallampati grading I and II requiring elective orotracheal intubation for mechanical ventilation in the ICU were included in the study [17]. Patients with reduced mouth opening (less than 5 cm), Mallampati grading III and IV, traumatic injury to the face, unstable cervical spine, any contraindication to orotracheal intubation, and those who required a third attempt at intubation were excluded. Patients requiring urgent airway access and those whose relatives refused participation in the study were also excluded.

Methodology

The patients were randomly allocated into two groups. Group A patients were intubated with a Macintosh blade, and group B patients were intubated with a VL (BPL VL-02). All patients were intubated by the primary investigator who had done at least 30 successful intubations previously with each of these laryngoscopes. Preoxygenation was done using a bag-mask-valve for three minutes. Induction was done with injection propofol 2-2.5 mg/kg IV or etomidate 0.2-0.3 mg/kg IV and injection succinylcholine 1-1.5 mg/kg IV or alternatively with injection rocuronium bromide 1 mg/kg IV (where succinylcholine was contraindicated).

The following parameters were recorded by the assistant: the total number of intubation attempts (only two attempts were allowed to the primary investigator, patients requiring a third intubation attempt were excluded), total duration of intubation (time taken from the insertion of the laryngoscope in the oral cavity to successful placement of the endotracheal tube and confirmation with end-tidal CO_2_ till six breaths), the requirement of alternative techniques or assistance for intubation, Cormack-Lehane grading, and complications encountered during intubation. Reinsertion of the laryngoscope into the oral cavity was considered another attempt. After two unsuccessful attempts or in case of a decrease in SpO_2_ to less than 90% or any other complications (bleeding, trauma), the help of the senior-most team member was sought.

Statistical analysis

Statistical analysis was performed using SPSS software for Windows version 25.0 (IBM Corp., Armonk, NY, USA). Continuous variables were evaluated by mean (standard deviation) or range when required. Dichotomous variables were presented as number/frequency and were analyzed using the chi-square test. For comparison of the means between the two groups, analysis by Student’s t-test with a 95% confidence interval was used. A p-value <0.05 was considered significant.

## Results

**Figure 1:**
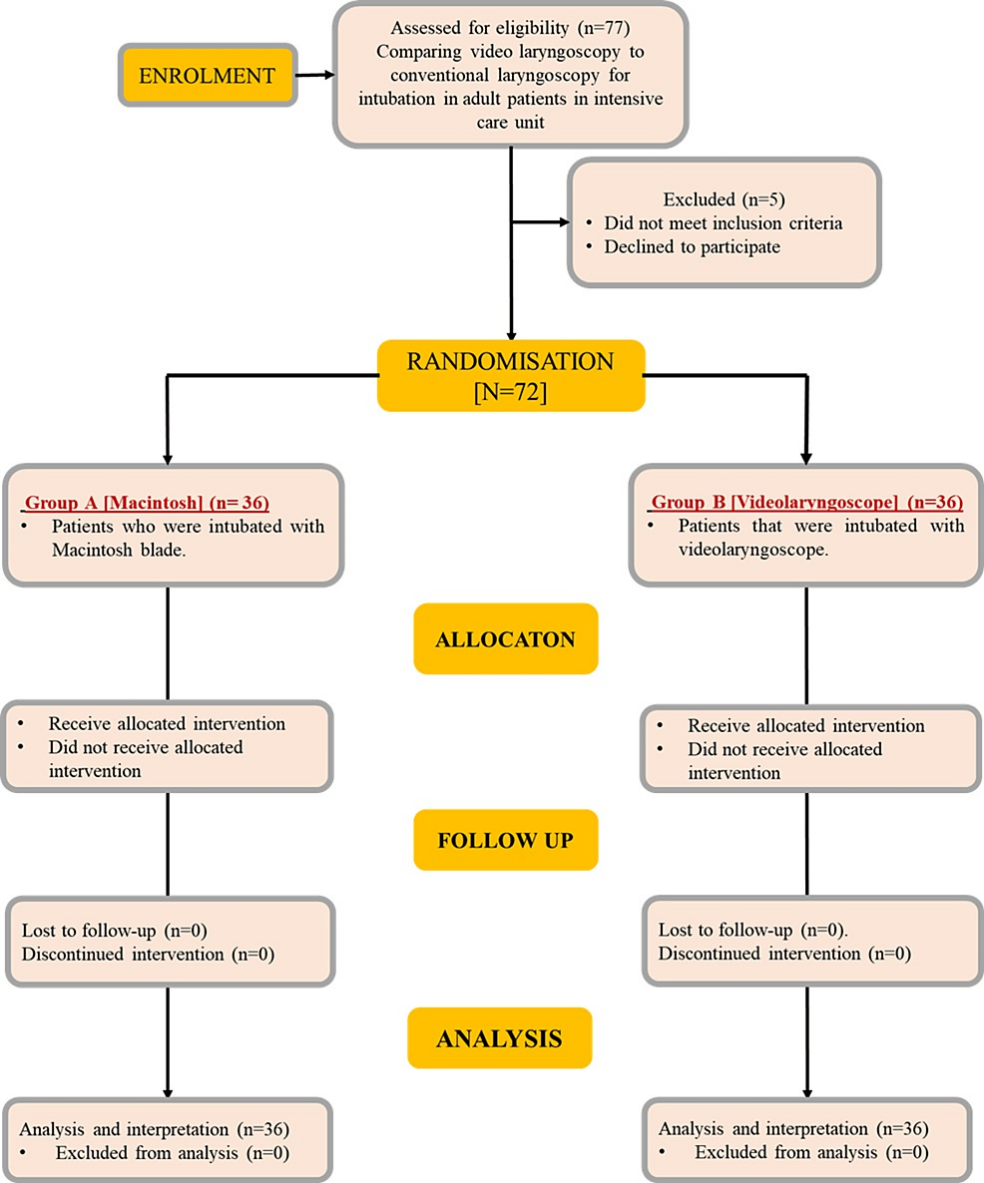
Flowchart showing participant selection.

Figure 1 shows the flowchart of patient selection. On comparing the demographic data of the two groups, it was found that the mean age, weight, and height of the patients were comparable between the two groups. Most patients in both groups were between the age group of 18 and 30 years. On comparing the gender of patients between the groups, the distribution of males and females was found to be similar (Table 2).

**Table 2:** Demographic profile of the study participants.

Demographic profile	Group A (N = 36)		Group B (N = 36)		P-value
	Mean	SD	Mean	SD	
Age	35.17	13.70	38.75	14.03	t = 1.095, p = 0.2771
Gender					
Female	17	47.22%	16	44.44%	X = 0.05594, p = 0.8130
Male	19	52.78%	20	55.56%	
Weight	61.39	9.90	60.39	8.85	t = 0.4518, p = 0.6528
Height	165.39	5.46	165.67	4.92	t = 0.2286, p = 0.8199

For assessing the airway, Mallampati grading was done. It was observed that the maximum number of patients belonged to grade 1 in both group A (23 (41.67%)) and group B (28 (77.78%)). Statistically, no significant difference was found between the groups (p = 0.1948) (Table 3).

**Table 3:** Airway assessment of the study participants in the two groups.

Mallampati grading	Group A (N = 36)		Group B (N = 36)		P-value
	N	%	N	%	
1	23	41.67%	28	77.78%	X = 1.681, p = 0.1948
2	13	58.33%	8	22.22%	

There was no significant difference in the Cormack-Lehane grading of patients in the two groups. The maximum number of patients belonged to grade 1 in both group A (21 (58.33%)) and group B (24 (66.67%)). Statistically, no significant difference (p = 0.4652) was noted between the groups.

On comparing the number of intubation attempts on patients, it was found that 30 (83.33%) patients from group A could be intubated in only one attempt compared to 26 (72.22%) patients in group B. Moreover, six (16.67%) patients in group A were intubated in the second attempt compared to 10 (27.88%) in group B. However, the differences were non-significant (p = 0.7053 and 0.4760) (Table 4).

**Table 4:** Percentage of successful first-pass and second-attempt intubations.

Number of intubation attempts	Group A (N = 36)		Group B (N = 36)		P-value
	N	%	N	%	
1	30	83.30%	26	72.22%	X = 0.143, p = 0.7053
2	6	16.67%	10	27.88%	X = 0.5079, p = 0.4760

**Figure 2:**
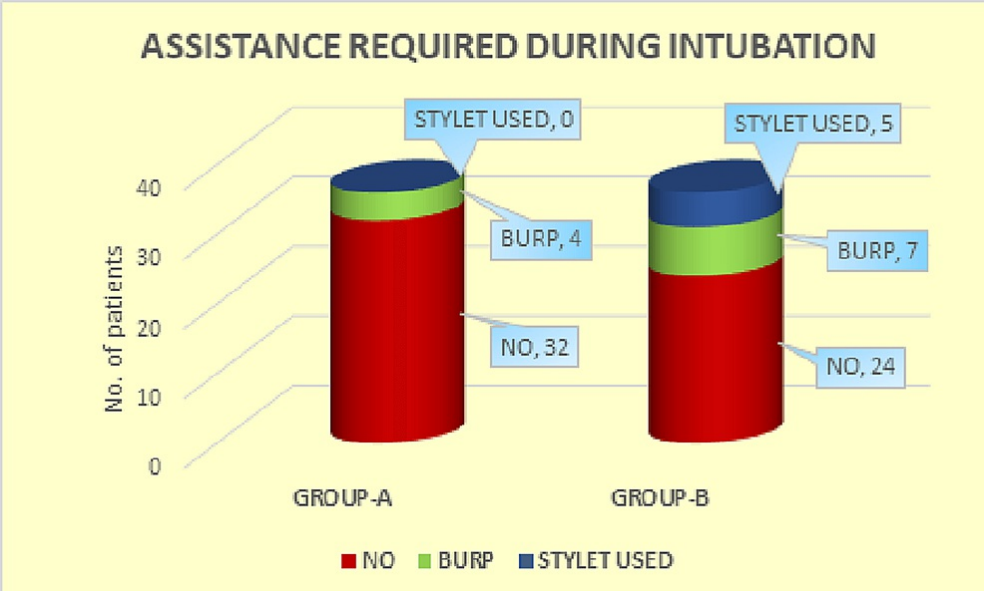
Graphical representation of the comparison of assistance required during intubation in patients between the groups. BURP: backward, upward, rightward, and posterior pressure on the larynx

On comparing the assistance required during intubation in patients, it was observed that the maximum number of patients in both groups required no assistance during intubation. Four (11.11%) patients in group A and seven (19.44%) patients in group B needed backward, upward, and rightward pressure on the larynx during intubation. It was further noted that in five (13.89%) patients in group B, a Stylet was used during intubation. A significant difference (p = 0.0308) was observed in assistance required during intubation between the two groups (Table 5, Figure 2).

**Table 5:** Comparison of assistance required during intubation. BURP: backward, upward, rightward, and posterior pressure on the larynx

Assistance required during intubation	Group A (N = 36)		Group B (N = 36)		P-value
	N	%	N	%	
No	32	88.89%	24	66.67%	X = 6.961, p = 0.0308*
BURP	4	11.11%	7	19.44%	
Stylet used	0	0.00%	5	13.89%	

**Figure 3:**
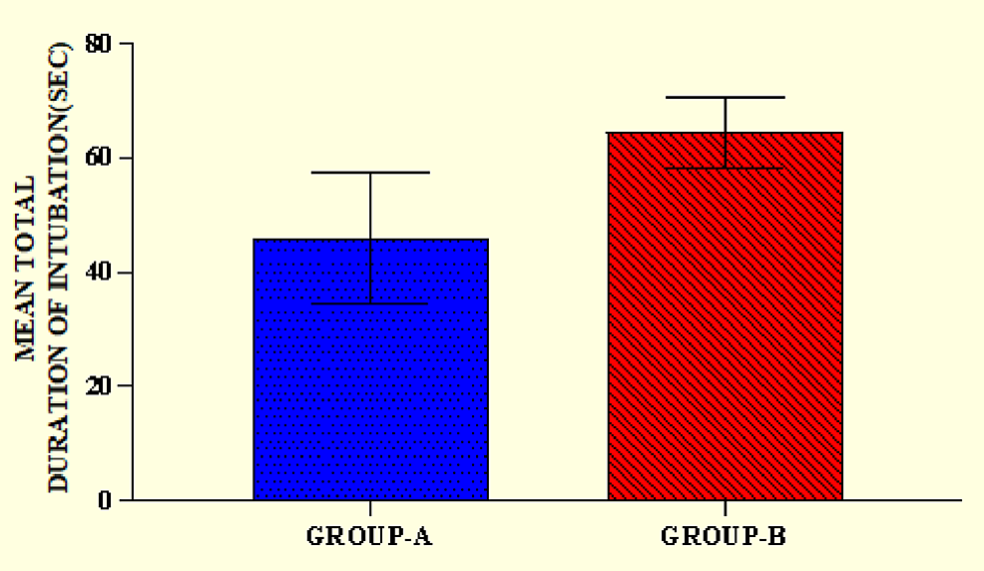
Mean total duration (or time taken) of intubations.

The mean time taken for intubation or the mean total duration of intubation was calculated. Group B had a longer duration of intubation (64.36 ± 6.28 seconds) compared to group A (45.72 ± 11.45 seconds), with a statistically significant difference among them (p < 0.0001) (Figure 3).

It was observed that in two patients in group A and one patient in group B, trauma to the oral cavity occurred during intubation. One patient in each group developed hypoxemia (SpO_2_ <90%). In two patients in group A, the endotracheal tube went into the esophagus in the first attempt. However, there was no significant difference (p = 0.6908) in overall complications among the two groups.

## Discussion

VLs have been shown to improve the laryngeal view [8], although in our study the Cormack-Lehane grading did not differ significantly between the two groups (p = 0.4652). In addition, there was no significant difference in the successful first-pass intubations between the two groups (p = 0.7053). We observed that more patients in the VL group required a second attempt at intubation compared to the CL group, but the difference was not statistically significant (p = 0.476). More assistance was required during the intubation of patients in the VL group in the form of BURP maneuver and Stylet. The total duration of intubation was also longer in the VL group. Significant life-threatening complications were not reported in any of the groups.

VLs may improve the success rate of tracheal intubation due to improved laryngeal view, although the optimal device for specific patients and groups varies. In genuinely difficult cases, intubation time may be prolonged [18], as was seen in our study too. It has also been demonstrated that VL reduces hemodynamic reactions to tracheal intubation, as well as the pressures of intubation and the pressure exerted over teeth, potentially reducing dental trauma [19-21]. This can be particularly helpful in suspected cervical spine injuries and trauma cases. But the grade 1 Cormack-Lehane view does not necessarily guarantee a successful tracheal tube insertion. Studies have demonstrated no significant difference in successful first-pass intubations between VL and CL groups despite a better view in the VL group [22,23]. Moreover, a Stylet is often required to negotiate the oropharyngeal angle and facilitate the passage of the endotracheal tube. We also observed that more assistance was required with a VL than with a CL. A study showed that the use of a gum-elastic bougie and/or external laryngeal manipulation was required more often in the C-MAC intubations compared with direct laryngoscopy [10]. A study by Klabusayova et al. in 2021 also reported longer intubation time with VL similar to our study [24]. Kleine-Brueggeney et al. [6] suggested that VL performance may be situation-specific; hence, they opined that generalizing the results to other patients is not justified as it will cause significant implications for future research. Recent systematic reviews and meta-analyses have shown that VL provides improved views of the glottis compared to CL and can be utilized to control troublesome airways [23]. However, comparative analyses of the various forms of VL have only been undertaken in a small number of instances [25].

A study by Chew et al. showed a better success rate with channeled VL blades compared to non-channeled blades [26]. The use of non-channeled VL blades in our study may be the reason for the difficulty encountered by us during intubation. Also, our study included only those patients who required elective endotracheal intubation in the ICU, and hence, the findings may not be generalized to all settings. The results were limited to a single tertiary care center. Hence, it cannot be extrapolated to a larger population. Only one type of VL blade was compared in our study. These can be considered as limitations of this study.

## Conclusions

From the findings of our study, we can conclude that a non-channeled VL (BPL VL-02) is not a suitable alternative to conventional direct laryngoscopy with a Macintosh blade in terms of successful first-pass intubation and total duration of intubation when used for adult patients in the ICU requiring elective orotracheal intubation for mechanical ventilation. Although the glottic visualization is comparable to the CL, more assistance and more time are required for intubation with VL. However, more recommendations can be made after conducting a multicentric study with a high descriptive sample size in the future. Additionally, periodic surveys should be done for any change or update in the pattern of results.

Many manufacturers are producing VLs with differing specifications, user interfaces, and geometry. All offer better glottic visualization but the number of attempts necessary to intubate the trachea and average intubation time may vary significantly among different VLs. Therefore, we cannot pass the same verdict for all types of VLs. Intubation can be easier with channeled blades compared to non-channeled blades which may be the reason for the longer intubation time and assistance required for intubation in our study.

## Figures and Tables

**Figure 4:**
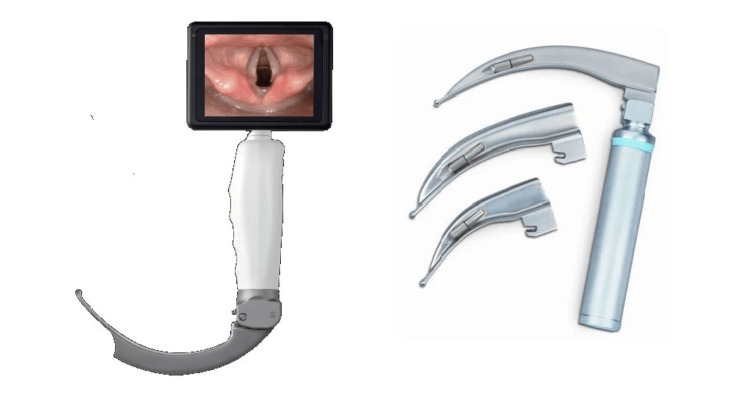
Image showing the video laryngoscope BPL VL-02 and the conventional direct laryngoscope.

## References

[1] Alvarado AC, Panakos P (2022). Endotracheal Tube Intubation Techniques. http://www.ncbi.nlm.nih.gov/books/NBK560730/.

[2] Mechlin MW, Hurford WE (2014). Emergency tracheal intubation: techniques and outcomes. Respir Care.

[3] Hsiao YJ, Chen CY, Hung HT, Lee CH, Su YY, Ng CJ, Chou AH (2021). Comparison of the outcome of emergency endotracheal intubation in the general ward, intensive care unit and emergency department. Biomed J.

[4] Cook T, Behringer EC, Benger J (2012). Airway management outside the operating room: hazardous and incompletely studied. Curr Opin Anaesthesiol.

[5] Cavus E, Byhahn C, Dörges V (2017). Classification of videolaryngoscopes is crucial. Br J Anaesth.

[6] Kleine-Brueggeney M, Greif R, Schoettker P, Savoldelli GL, Nabecker S, Theiler LG (2016). Evaluation of six videolaryngoscopes in 720 patients with a simulated difficult airway: a multicentre randomized controlled trial. Br J Anaesth.

[7] Berkow LC, Morey TE, Urdaneta F (2018). The technology of video laryngoscopy. Anesth Analg.

[8] Choudhary J, Barai AK, Das S, Mukherjee N (2021). Evaluation of the use of the channeled King Vision video laryngoscope in improving glottic visualisation in patients with limited glottic view with the Macintosh laryngoscope: a prospective observational study. Indian J Anaesth.

[9] Patel N, Desai DJ (2021). Tracheal intubation with King Vision video laryngoscope in patients with cervical spine instability-comparison of straight versus curved reinforced endotracheal tubes. Indian J Anaesth.

[10] Aziz MF, Kim D, Mako J, Hand K, Brambrink AM (2012). A retrospective study of the performance of video laryngoscopy in an obstetric unit. Anesth Analg.

[11] Grunwell JR, Kamat PP, Miksa M (2017). Trend and outcomes of video laryngoscope use across PICUs. Pediatr Crit Care Med.

[12] De Jong A, Pardo E, Rolle A, Bodin-Lario S, Pouzeratte Y, Jaber S (2020). Airway management for COVID-19: a move towards universal videolaryngoscope?. Lancet Respir Med.

[13] Hamal PK, Chaurasia RB, Pokhrel N, Pandey D, Shrestha GS (2020). An affordable videolaryngoscope for use during the COVID-19 pandemic. Lancet Glob Health.

[14] Cormack RS, Lehane J (1984). Difficult tracheal intubation in obstetrics. Anaesthesia.

[15] Yi IK, Hwang J, Min SK, Lim GM, Chae YJ (2021). Comparison of learning direct laryngoscopy using a McGrath videolaryngoscope as a direct versus indirect laryngoscope: a randomized controlled trial. J Int Med Res.

[16] Fleiss JL, Tytun A, Ury HK (1980). A simple approximation for calculating sample sizes for comparing independent proportions. Biometrics.

[17] Mallampati SR, Gatt SP, Gugino LD, Desai SP, Waraksa B, Freiberger D, Liu PL (1985). A clinical sign to predict difficult tracheal intubation: a prospective study. Can Anaesth Soc J.

[18] Norris A, Heidegger T (2016). Limitations of videolaryngoscopy. Br J Anaesth.

[19] Hinkelbein J, Iovino I, De Robertis E, Kranke P (2019). Outcomes in video laryngoscopy studies from 2007 to 2017: systematic review and analysis of primary and secondary endpoints for a core set of outcomes in video laryngoscopy research. BMC Anesthesiol.

[20] Kriege M, Alflen C, Tzanova I, Schmidtmann I, Piepho T, Noppens RR (2017). Evaluation of the McGrath MAC and Macintosh laryngoscope for tracheal intubation in 2000 patients undergoing general anaesthesia: the randomised multicentre EMMA trial study protocol. BMJ Open.

[21] Ruetzler K, Rivas E, Cohen B (2020). McGrath video laryngoscope versus Macintosh direct laryngoscopy for intubation of morbidly obese patients: a randomized trial. Anesth Analg.

[22] Lascarrou JB, Boisrame-Helms J, Bailly A (2017). Video laryngoscopy vs direct laryngoscopy on successful first-pass orotracheal intubation among ICU patients: a randomized clinical trial. JAMA.

[23] Risse J, Volberg C, Kratz T, Plöger B, Jerrentrup A, Pabst D, Kill C (2020). Comparison of videolaryngoscopy and direct laryngoscopy by German paramedics during out-of-hospital cardiopulmonary resuscitation; an observational prospective study. BMC Emerg Med.

[24] Klabusayová E, Klučka J, Kosinová M (2021). Videolaryngoscopy vs. direct laryngoscopy for elective airway management in paediatric anaesthesia: a prospective randomised controlled trial. Eur J Anaesthesiol.

[25] Healy DW, Maties O, Hovord D, Kheterpal S (2012). A systematic review of the role of videolaryngoscopy in successful orotracheal intubation. BMC Anesthesiol.

[26] Chew SH, Lim JZ, Chin BZ, Chan JX, Siew RC (2019). Intubation with channeled versus non-channeled video laryngoscopes in simulated difficult airway by junior doctors in an out-of-hospital setting: a crossover manikin study. PLoS One.

